# SETDB1 regulates microtubule dynamics

**DOI:** 10.1111/cpr.13348

**Published:** 2022-11-03

**Authors:** Rosari Hernandez‐Vicens, Jagreeti Singh, Nomi Pernicone, Tamar Listovsky, Gabi Gerlitz

**Affiliations:** ^1^ Department of Molecular Biology, Faculty of Life Sciences Ariel University Ariel Israel; ^2^ Ariel Center for Applied Cancer Research Ariel University Ariel Israel; ^3^ Adelson School of Medicine Ariel University Ariel Israel

## Abstract

**Objectives:**

SETDB1 is a methyltransferase responsible for the methylation of histone H3‐lysine‐9, which is mainly related to heterochromatin formation. SETDB1 is overexpressed in various cancer types and is associated with an aggressive phenotype. In agreement with its activity, it mainly exhibits a nuclear localization; however, in several cell types a cytoplasmic localization was reported. Here we looked for cytoplasmic functions of SETDB1.

**Methods:**

SETDB1 association with microtubules was detected by immunofluorescence and co‐sedimentation. Microtubule dynamics were analysed during recovery from nocodazole treatment and by tracking microtubule plus‐ends in live cells. Live cell imaging was used to study mitotic kinetics and protein–protein interaction was identified by co‐immunoprecipitation.

**Results:**

SETDB1 co‐sedimented with microtubules and partially colocalized with microtubules. SETDB1 partial silencing led to faster polymerization and reduced rate of catastrophe events of microtubules in parallel to reduced proliferation rate and slower mitotic kinetics. Interestingly, over‐expression of either wild‐type or catalytic dead SETDB1 altered microtubule polymerization rate to the same extent, suggesting that SETDB1 may affect microtubule dynamics by a methylation‐independent mechanism. Moreover, SETDB1 co‐immunoprecipitated with HDAC6 and tubulin acetylation levels were increased upon silencing of SETDB1.

**Conclusions:**

Taken together, our study suggests a model in which SETDB1 affects microtubule dynamics by interacting with both microtubules and HDAC6 to enhance tubulin deacetylation. Overall, our results suggest a novel cytoplasmic role for SETDB1 in the regulation of microtubule dynamics.

## Graphical abstract

SETDB1 association with microtubules inhibits microtubule polymerization and enhances their instability. SETDB1 may affect the microtubules by interacting with HDAC6 to enhance HDAC6 tubulin deacetylation activity.

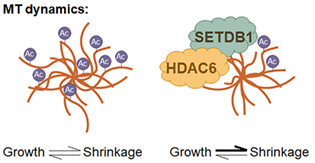

## 1 INTRODUCTION

SETDB1 (also known as ESET and KMT1E) is a lysine methyltransferase (KMT) that belongs to the suppressor of variegation 3–9 (SUV39) family of KMTs that mainly methylate lysine 9 in histone H3 (H3K9). The SUV39 family members are characterized by cysteine‐rich pre‐ and post‐SET domains flanking a central SET (Su(var)3–9, Enhancer‐of‐zeste and Trithorax) domain that is responsible for the catalytic activity.^1^, ^2^ SETDB1 also contains a methyl‐CpG‐binding domain (MBD) and a triple Tudor domain responsible for binding of H3K9me/K14ac to promote histone deacetylation by histone deacetylases (HDACs).^3^, ^4^ SETDB1 can mono‐, di‐ and tri‐methylate H3K9. H3K9me2/3 are usually associated with gene repression and heterochromatin formation,^1^ and indeed SETDB1 is involved in silencing the X chromosome,^5^, ^6^ repetitive elements^7^, ^8^, ^9^, ^10^ and specific genes.^11^, ^12^, ^13^ SETDB1 is important for various developmental processes including embryogenesis,^14^, ^15^ neurogenesis,^16^ immune cell development^17^ and germ line development.^8^ SETDB1 was also shown to methylate non‐histone proteins such as inhibitor of growth protein 2 (ING2),^18^ p53^19^ and upstream binding factor (UBF).^20^

SETDB1 is considered a nuclear protein; and indeed, its substrates either histone H3 or non‐histone proteins, are mainly nuclear proteins. However, SETDB1 contains both nuclear localization signal (NLS) motifs and nuclear export signal (NES) motifs.^21^, ^22^ In addition, a cytoplasmic pool of SETDB1 has been found in several types of cells including HeLa cells,^23^ HEK293 cells,^24^ mouse embryonic fibroblasts,^22^ differentiated myoblasts^21^ and human melanoma biopsies.^25^ Cytoplasmic localization of SETDB1 is thought to facilitate methylation of newly synthesized histones before their incorporation into nucleosomes^26^ or to restrict the enzyme of methylating nucleosomal H3K9.^21^, ^22^

In recent years, SETDB1 has been considered an oncogene. Its genomic location is commonly amplified in melanoma^27^, ^28^ and its expression levels are increased in various types of cancer to support cancer cell proliferation, migration and invasion. These types of cancer include melanoma,^27^, ^28^ colorectal cancer,^29^, ^30^ liver cancer^19^, ^31^ and lung cancer.^32^, ^33^ More recently, SETDB1 was also linked to adaptive resistance of tumour cells to various drugs^2^ and repression of the innate immune response.^7^, ^34^

Currently, SETDB1 is thought to promote cancer mainly by its nuclear activity of methylating H3K9 or transcription factors such as p53.^19^ However, since a substantial amount of SETDB1 can be found in the cytoplasm, we looked for cytoplasmic activity of SETDB1 that may also promote cancer formation and progression. Here we show that SETDB1 co‐sedimented with microtubules (MTs) and that cytoplasmic SETDB1 partially co‐localized with the MT network. Reduced SETDB1 levels increased MT stability as measured by EB1‐tracking and MT recovery from nocodazole treatment and interfered with mitotic progression. Over‐expression of either wild type (WT) or catalytic dead (CD) SETDB1 increased MT polymerization rate to the same extent, suggesting that SETDB1 can affect MT dynamics by an additional mechanism to substrate methylation. Finding interaction between SETDB1 and the tubulin deacetylase histone deacetylase 6 (HDAC6), along with increased tubulin acetylation levels after reduction in SETDB1 levels, suggest that SETDB1 may affect MT dynamics by supporting HDAC6 activity.

## 2 MATERIALS AND METHODS

### 2.1 Plasmids and molecular cloning

Plasmids expressing GFP‐fused WT and H1224K SETDB1 fused to GFP were generated by PCR using pREV‐SETDB1 as a template (a kind gift from Slimane Ait‐Si‐Ali, Centre National de la Recherche Scientifique [CNRS], Université de Paris, Université Paris Diderot, Paris, France).^35^ WT SETDB1 was amplified by KOD Hot Start DNA Polymerase (71086, Merck) and the oligonucleotides 5′‐cagagctcATGTCTTCCCTTCCTG GGTGCAT‐3′ and 5′‐gtgtcgaCTAAAGAAGACGTCCTCTGCATTCAAT‐3′. The PCR product was ligated into SacI‐SalI sites in pEGFP‐C3. Site‐directed mutagenesis to generate SETDB1 H1224K was performed by PCR using the above two oligonucleotides and the oligonucleotides: 5′‐GGGCCGCTACCTCAACaagAGTTGCAGCCCCAACC‐3′ and 5′‐GGTTGGGGCTGC AACTcttGTTGAGGTAGCGGCCC‐3′. Cloning procedures were confirmed by DNA sequencing. pcDNA‐HDAC6‐FLAG was a gift from Tso‐Pang Yao (Addgene plasmid # 30482).^36^ EB1‐GFP was a kind gift from Orly Reiner, Weizmann Institute of Science, Rehovot, Israel. pEGFP‐Moesin was a kind gift from Peter Vilmos, Biological Research Center of the Hungarian Academy of Sciences, Szeged, Hungary.

### 2.2 Cell culture, transfections and proliferation assay

B16‐F1 cells (ATCC, CRL‐6323), WM266.4 cells (a kind gift from Gal Markel, Sheba Medical Center, Israel), HeLa cells (ATCC, CCL‐2) and HEK293 cells (ATCC, CRL‐1573) were cultured in DMEM (Biological Industries, Kibbutz Beit‐Haemek, Israel) supplemented with 10% fetal calf serum, 0.5% penicillin ‐ streptomycin solution mix, and 1% l‐glutamine, at 37°C in a 7% CO_2_ environment. Transfections of DNA plasmids were carried out with the NanoJuice Transfection Kit (71900‐3, Merck, Kenilworth, NJ, USA) or the Avalanche‐Everyday Transfection Reagent (EZT‐EVDY‐1, EZ Biosystems, College Park, MD, USA) according to the manufacturers' instructions. Cells were incubated for 24 h before further analysis. For gene silencing, cells were transfected twice with siRNA (IDT, Coralville, IA, USA), with a time interval of 48 h using INTERFERin transfection reagent (Polyplus‐transfection, Illkirch‐Graffenstaden, France) according to the manufacturer's instructions. Cells were incubated for 24 h after the second transfection before further analysis. SiRNAs used were mouse SETDB1 (mm.Ri.Setdb1.13.1), human SETDB1 (hs.Ri.SETDB1.13.1) and negative control (51‐01‐14‐04). SiRNA transfection efficiency was >90% as verified by transfection of Cy3 Transfection Control DsiRNA (51‐01‐03‐06). Proliferation rate was measured with Cell Proliferation Kit (XTT based) (20‐300‐1000, Biological Industries, Kibbutz Beit‐Haemek, Israel), according to the manufacturer's protocol.

### 2.3 Protein lysate preparation and Western blot analysis

For nuclear–cytoplasmic fractionation, cells were washed in PBS, re‐suspended in STM buffer: 50 mM Tris pH 7.4, 250 mM sucrose, 5 mM MgSO_4_, 10 mM NaF, 2 mM DTT, 0.025% Triton X‐100 and 1× protease inhibitor cocktail (539134, Millipore, Burlington, MA, USA) and lysed by a Dounce homogenizer. Following cell membrane breakage (as monitored under the microscope), the nuclei fraction was precipitated at 700 *g* for 10 min at 4°C. The cytoplasmic supernatant was collected to a new tube and the nuclear pellet was re‐suspended in TP buffer: 10 mM Tris pH 7.4, 10 mM phosphate buffer pH 7.4, 5 mM MgSO_4_, 10 mM NaF and 1× protease inhibitor cocktail supplemented with 5% glycerol. Protein concentrations were measured using the Bradford assay. Samples were stored at −80°C. For whole cell extracts, cells were washed in PBS, re‐suspended in 2× SDS sample buffer (100 mM Tris pH 6.8, 10% glycerol, 2% SDS, 100 mM bromophenol blue, 0.1 M DTT and 1× protease inhibitor cocktail) and sonicated. Samples were then heated at 95°C for 10 min and kept at −20°C until use.

Protein extracts were separated in SDS‐PAGE and analysed by Western blot analysis using the following antibodies: rabbit anti‐SETDB1 (1:500, Santa Cruz Biotechnology sc‐66884), rabbit anti‐SUV39H2 (1:1000, Abcam ab190270), rabbit anti‐histone H3 (1:10,000, Millipore 05‐928), mouse anti‐α‐tubulin (1:5,000, Thermo Fisher Scientific 62204), mouse anti‐acetylated tubulin (1:1000, Santa Cruz Biotechnology sc‐23950), mouse anti‐β‐actin (1:5000, Sigma‐Aldrich A1978), mouse anti‐GFP (1:500, Santa Cruz Biotechnology sc‐9996) and rabbit anti‐FLAG (1:1000, Sigma‐Aldrich F7425). Where indicated, quantitative data analysis was performed with the ImageJ/Fiji software (National Institute of Health, Bethesda, USA) using three repetitions and statistical significance was calculated with the Student's *t‐*test.

### 2.4 MT co‐sedimentation assay

MT co‐sedimentation assay was performed as reported previously.^37^ Briefly, cells were lysed in PIPES buffer: 80 mM PIPES pH 6.8, 1 mM MgCl_2_, 1 mM EGTA, 100 mM NaCl, 1% Triton X‐100, and 1× protease inhibitor cocktail (539134, Millipore, Burlington, MA, USA) for 30 min on ice. Cell lysates were cleared by two repetitive centrifugations at 20,000*g* for 20 min at 4°C. The supernatants supplemented with 1 mM GTP and 40 μM Paclitaxel were incubated at 4°C or 37°C for 30 min for tubulin polymerization before centrifugation at 20,000*g* for 30 min at 4°C or 37°C, respectively. The resulting pellets and supernatants were subject to Western blot analysis.

### 2.5 Immunostaining

Cells plated on fibronectin‐coated coverslips (03‐090‐1‐05, Biological Industries, Beit‐Haemek, Israel) were fixed in methanol supplemented with 1 mM EGTA at −20°C for 6 min. Antibodies included rabbit anti‐SETDB1 (1:50, Santa Cruz Biotechnology sc‐66884), rabbit anti‐SETDB1(1:500, Cell Signaling Technology 93212), rabbit anti‐SUV39H2 (1:120, Abcam ab190270), mouse anti‐α‐tubulin (1:200, Thermo Fisher Scientific 62204), goat anti‐GFP (1:2000, Abcam ab5450) and rabbit anti‐γ‐tubulin (1:400, Abcam ab1132). All images were collected using an Olympus 1X81 fluorescent microscope with a coolSNAP HQ2 CCD camera (Photometrics, Tuscon AZ, USA).

### 2.6 MT recovery assay

Cells plated on fibronectin‐coated coverslips were treated with 7 μg/ml of nocodazole for 3 h. Following three washings with cold DMEM to remove the nocodazole, the cells were incubated at 37°C in pre‐warmed complete growth medium for the indicated periods of time, fixed and immunostained as described above. Quantitative data analysis was performed with ImageJ/Fiji software (NHI) by manual delineation of the area covered by MTOC‐linked MTs. At least 30 cells were measured for each condition in each repetition and the average size of treated cells was normalized to the one of control cells. Average scores of three repetitions were calculated and statistical significance was determined by the Student's t‐test.

### 2.7 Time‐lapse imaging

For live‐cell imaging, cells were plated in 35‐mm glass‐bottom dishes. Time‐lapse images were collected with a coolSNAP HQ2 CCD camera (Photometrics, Tuscon, AZ, USA) mounted on an Olympus 1X81 fluorescent microscope at 37°C and 7% CO_2_. Frames were captured every 3 s for 1.5 min (movies to track growing MT ends) or every 3 min for 10 h (movies to monitor mitotic progression). Acquired images were analysed by ImageJ/Fiji software. To analyse growing MT ends, EB1‐GFP comets were tracked manually using the MTrackJ ImageJ plugin.^38^ Comets were analysed in each frame considering the distal site of the comet as the comet point. Data analysis was done according to duration and length tracked. To analyse mitosis progression, mitotic events were followed in terms of time and success rate.

### 2.8 Co‐immunoprecipitation

HEK293 cells were harvested 48 h following co‐transfection and lysed in extraction buffer (50 mM Tris pH 8, 150 mM NaCl, 20 mM EDTA, 50 mM NaF, 1% Triton X‐100) supplemented with 1× protease inhibitor cocktail. Cells were lysed on ice for 30 min and centrifuged at 20,000*g* for 30 min at 4°C. A 5% input control sample was taken from each cleared lysate and boiled in SDS sample buffer for 10 min at 98°C. For immunoprecipitation, clarified lysates were supplemented with GFP Trap Magnetic Agarose (gtma‐20, Chromotek, Planegg‐Martinsried, Germany) and incubated with rotation for 1 h at 4°C. The beads were washed once in extraction buffer and three times in PBS and boiled in SDS sample buffer for 10 min at 98°C, before being loaded on an 8% acrylamide gel for subsequent Western blot analysis.

## 3 RESULTS

### 3.1 SETDB1 associates with the MT network

**FIGURE 1:**
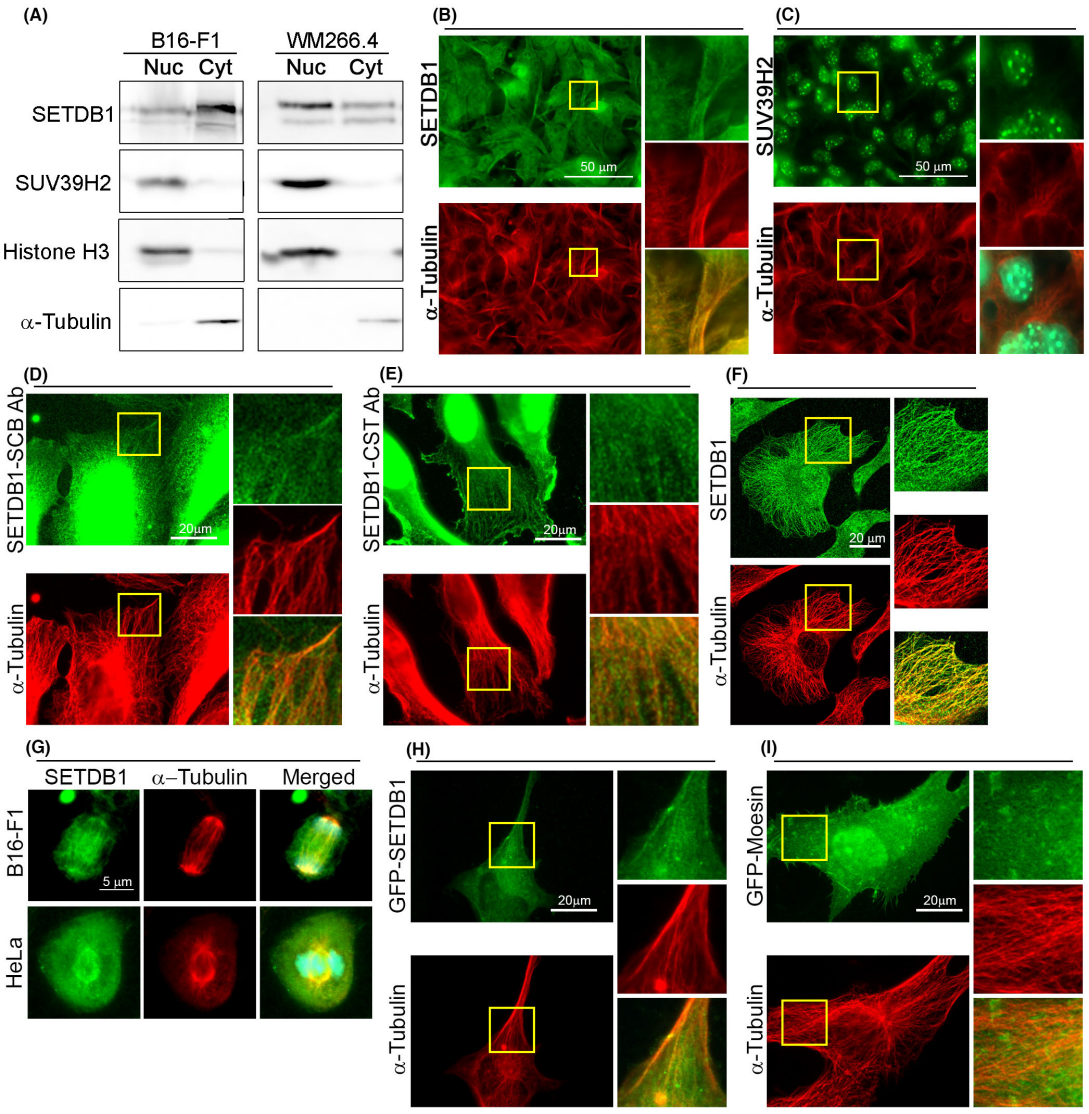
SETDB1 is found in the cytoplasm and partially co‐localizes with MTs. (A) Western blot analysis of nuclear (Nuc) and cytoplasmic (Cyt) fractions of mouse B16‐F1 cells and human WM266.4 cells for SETDB1, SUV39H2, α‐tubulin and histone H3. (B,C) B16‐F1 cells immunostained for SETDB1 or SUV39H2 and α‐tubulin. DNA stained with Hoechst 33342. Sections inside the inserts are magnified at the right side of each micrograph. The merged images show the merged signals of SETDB1, α‐tubulin and Hoechst. Scale bar: 50 μm. (D,E) WM266.4 cells immunostained for SETDB1 with two different commercial antibodies (SCB, Santa Cruz Biotechnology sc‐66884; CST, Cell Signaling Technology 93212) and α‐tubulin. Sections inside the inserts are magnified at the right side of each micrograph. The merged images show the merged signals of SETDB1 and α‐tubulin. Scale bar: 20 μm. (F) HeLa cells immunostained for SETDB1 and α‐tubulin. The merged images show the merged signals of SETDB1 and α‐tubulin. Scale bar: 20 μm. (G) Mitotic B16‐F1 cells and HeLa cells immunostained for SETDB1 and α‐tubulin. DNA stained with Hoechst 33342. The merged images show the merged signals of SETDB1, α‐tubulin and Hoechst. Scale bar: 5 μm. (H,I) WM266.4 cells transfected with either pEGFP‐SETDB1 or pEGFP‐Moesin immunostained for GFP and α‐tubulin. Sections inside the inserts are magnified at the right side of each micrograph. The merged images show the merged signals of GFP and α‐tubulin. Scale bar: 20 μm

**FIGURE 2:**
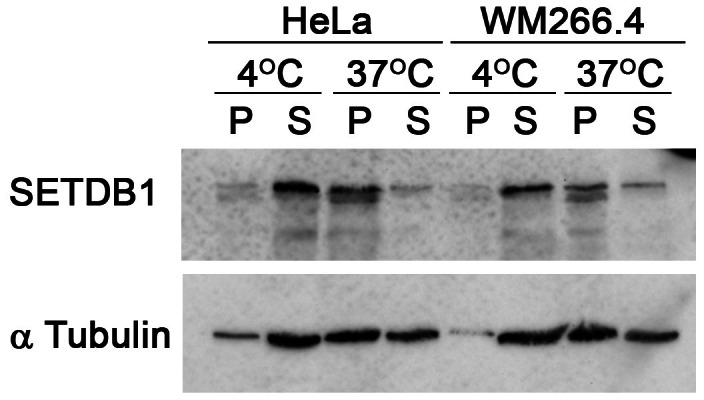
Co‐sedimentation of SETDB1 with MTs. MT co‐sedimentation assay of HeLa cells and WM266.4 cells. The pellet (P) of 37°C incubation indicates the MT‐bound fraction, and supernatant (S) indicates the unbound fraction. Incubation at 4°C serves as a MT‐free control

To identify a cytoplasmic role for SETDB1, we first verified its cytoplasmic localization in both mouse and human melanoma cells: B16‐F1 and WM266.4 cells, respectively. Biochemical fractionation followed by Western blot analysis identified a substantial amount of SETDB1 in the cytoplasmic fraction of these cells in contrast to another H3K9 methyltransferase, suppressor of variegation 3–9 homologue 2 (SUV39H2), which was found only in the nuclear fraction (Figure 1A). Immunostaining for SETDB1 verified this observation and revealed a partial co‐localization of SETDB1 with MTs in B16‐F1 cells (Figure 1B,C). In WM266.4 cells, the cytoplasmic fraction of SETDB1 was smaller than in B16‐F1 cells, though some co‐localization of cytoplasmic SETDB1 with MTs could still be detected (Figure 1D,E). To validate this observation, we used an additional antibody against SETDB1 that revealed a similar pattern of partial co‐localization (Figure 1D,E). Co‐localization of SETDB1 with MTs was also found in HeLa cells (Figure 1F). Moreover, this pattern was kept during mitosis in B16‐F1 cells and in HeLa cells (Figure 1G). Analysis of the localization of over‐expressed GFP‐fused SETDB1 revealed a similar pattern of partial co‐localization with MTs (Figure 1H,I). To verify this, we assessed MTs binding by co‐sedimentation. As shown in Figure 2, incubation of cell lysates at 37°C in conditions that promote MTs polymerization led to co‐sedimentation of SETDB1 in lysates prepared from HeLa and WM266.4 cells. Taken together, these results may suggest a role for SETDB1 in MT functions.

### 3.2 SETDB1 affects MT growth rate

**FIGURE 3:**
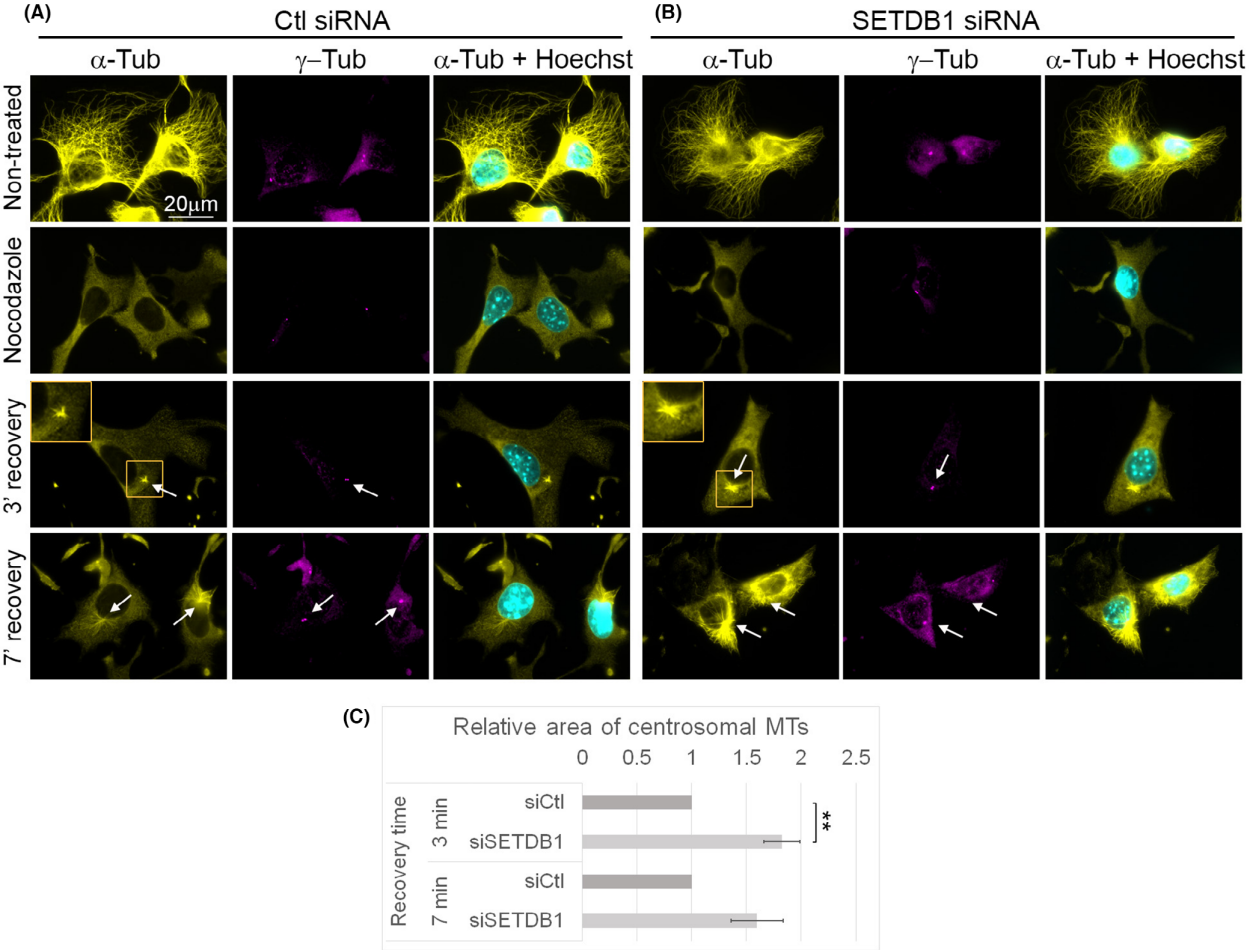
Reduced SETDB1 levels enhance MT polymerization rate. (A,B) MTs were disrupted in B16‐F1 cells transfected with either Ctl siRNA (A) or siRNA against SETDB1 (B) by nocodazole treatment for 3 h. Following nocodazole removal, cells were further incubated for the indicated time periods to allow MT polymerization. After fixation, cells were immunostained with antibodies against α‐tubulin, γ‐tubulin and DNA was stained with Hoechst 33342. The white arrows indicate the localization of the MTOCs in the recovery micrographs. The MTOCs in the orange rectangles are magnified at the left top corner of the micrographs of the 3 min of recovery from nocodazole treatment. The scale bar: 20 μm. (C) Quantification of MT recovery rate after nocodazole washout. The area covered by MTOC‐linked MTs was delineated manually and measured by ImageJ. The MT covered area in cells transfected with Ctl siRNA was set as 1. The bar graph shows the average relative area covered by MTs of three repetitions ±*SE*. At least 30 cells were measured for each condition in each repetition. Statistical significance was calculated with Student's *t‐*test, **p ˂ 0.01

To evaluate whether SETDB1 can affect MT functions, we first tested the impact of SETDB1 levels on the rate of MT growth during recovery from nocodazole treatment. B16‐F1 cells were transfected with control short interfering RNA (siRNA) or SETDB1 siRNA, which reduced SETDB1 levels by about 50%, probably due to its long half‐life (Figure S1). Then cells were treated with nocodazole for 3 h to depolymerize MTs. Following nocodazole washout, the cells were further incubated for an additional 3 or 7 min, fixed and immunostained for α‐tubulin. Measurements of the area covered by MTs originated from the MTOC (centrosomal MTs) revealed an increase of 82% and 60% in SETDB1 siRNA‐transfected cells, in comparison to control siRNA (Ctl siRNA)‐transfected cells at the 3 and 7 min time points, respectively (Figure 3). This finding suggests that SETDB1 may attenuate MT growth rate.

**FIGURE 4:**
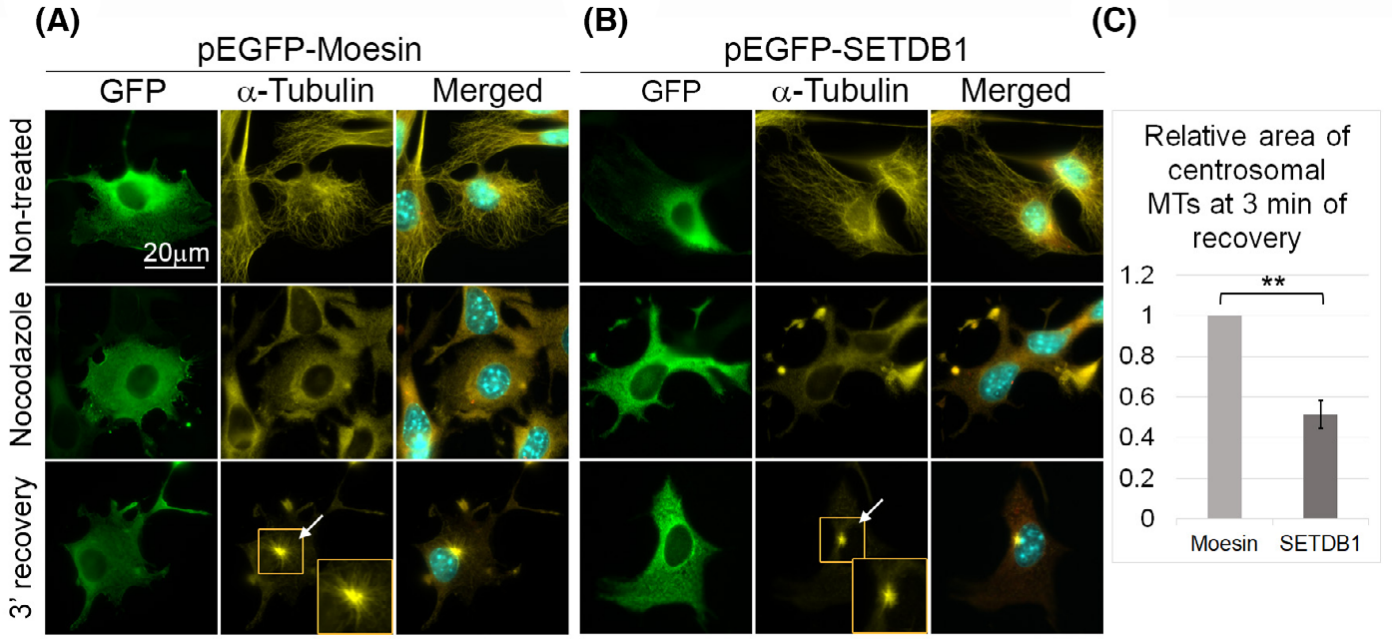
SETDB1 over‐expression enhances MT polymerization rate. (A,B) MTs were disrupted in B16‐F1 cells transfected with either pEGFP‐Moesin (A) or pEGFP‐SETDB1 (B) by nocodazole treatment for 3 h. Following nocodazole removal, cells were further incubated for 3 min to allow MT polymerization. After fixation, cells were immunostained with antibodies against GFP, α‐tubulin and γ‐tubulin and DNA was stained with Hoechst 33342. The white arrows indicate the localization of the MTOCs in the recovery micrographs. The MTOCs in the orange rectangles are magnified at the right bottom corner of the micrographs of recovery from nocodazole treatment. The merged images show the merged signals of α‐tubulin (yellow), γ‐tubulin (magenta) and Hoechst (cyan). Scale bar: 20 μm. (C) Quantification of MT recovery rate after nocodazole washout. The area covered by MTOC‐linked MTs was delineated manually and measured by ImageJ. The MT covered area in cells transfected with pEGFP‐Moesin was set as 1. The bar graph shows the average relative area covered by MTs of three repetitions ±*SE*. At least 30 cells were measured for each condition in each repetition. Statistical significance was calculated with Student's *t‐*test, **p ˂ 0.01

To verify the ability of SETDB1 to attenuate MT growth rate, we repeated the experiment in cells over‐expressing SETDB1. Interestingly, over‐expressed (OE) SETDB1 hardly accumulates in the nucleus and is localized mainly to the cytoplasm. This observation was reported before^22^, ^23^, ^39^ and repeated also in our hands (Figures 1H, 3B). For that experiment we used GFP‐Moesin as a negative control and found that the amount of MTOC‐linked MTs 3 min after nocodazole washout in GFP‐SETDB1 OE cells were reduced by half, compared to GFP‐Moesin OE cells (Figure 4). This effect was in the opposite direction to the effect of SETDB1 siRNA (Figure 3), strengthening the idea that SETDB1 attenuated MT growth rate.

**FIGURE 5:**
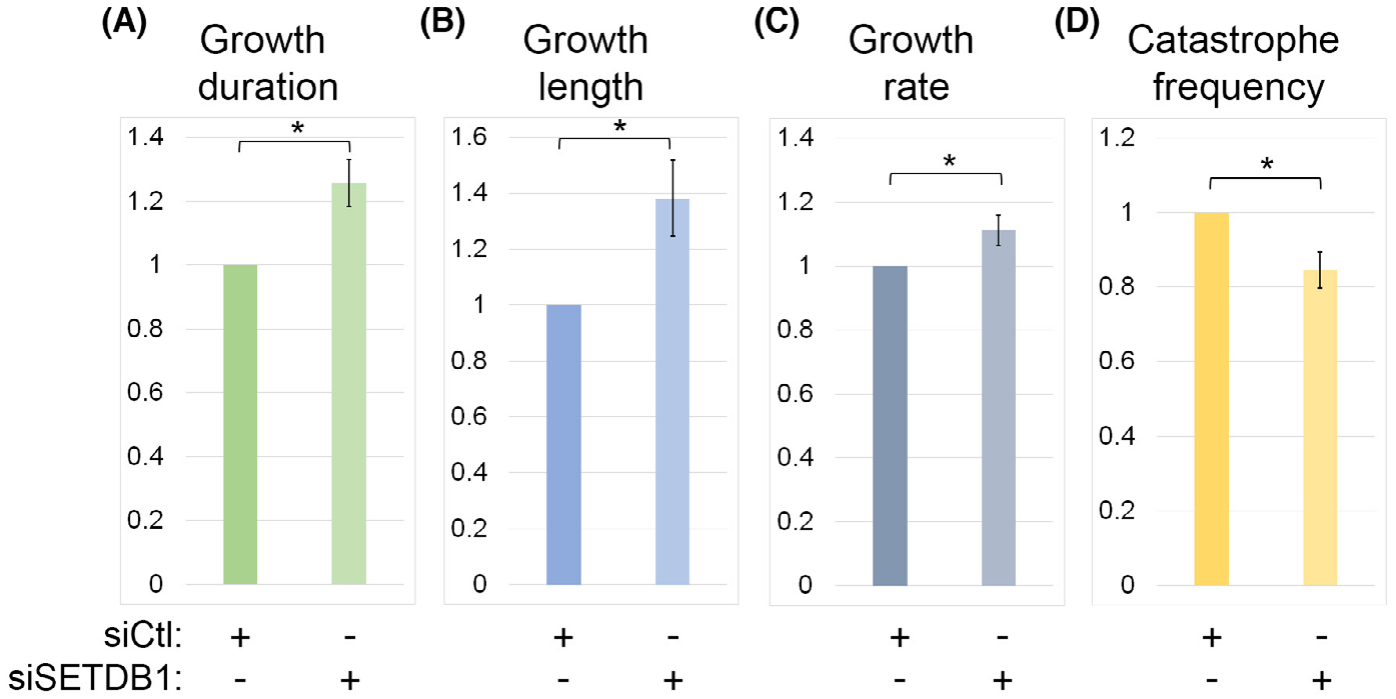
Reduced SETDB1 levels affect MT dynamics. (A–D) Parameters of MT dynamics as measured by EB1 tracking in HeLa cells by time‐lapse microscopy. The bar charts represent the ratios of the indicated parameters of MTs in SETDB1 siRNA transfected cells to Ctl siRNA transfected cells. The averages are of three repetitions ±*SE*. In each experiment a minimum of 100 EB1‐GFP comets per condition were measured. Statistical significance was calculated with Student's *t*‐test, *p ˂ 0.05

To validate the effects of SETDB1 on MT growth we monitored the MT plus end dynamics by tracking GFP‐fused end‐binding protein 1 (EB1) in SETDB1 siRNA‐transfected cells (Figures S1 and 5, Movies S1 and S2). Notably, MT growth duration, growth length and growth rate were increased in SETDB1 siRNA‐transfected cells by 25%, 38% and 11%, respectively, in comparison to control cells (Figure 5A–C). On the other hand, MT catastrophe rate in SETDB1 siRNA‐treated cells was reduced by 15% in comparison to control cells (Figure 5D). These results suggest that SETDB1 could be a negative regulator of MT growth.

### 3.3 SETDB1 is important for proper cell division

**FIGURE 6:**
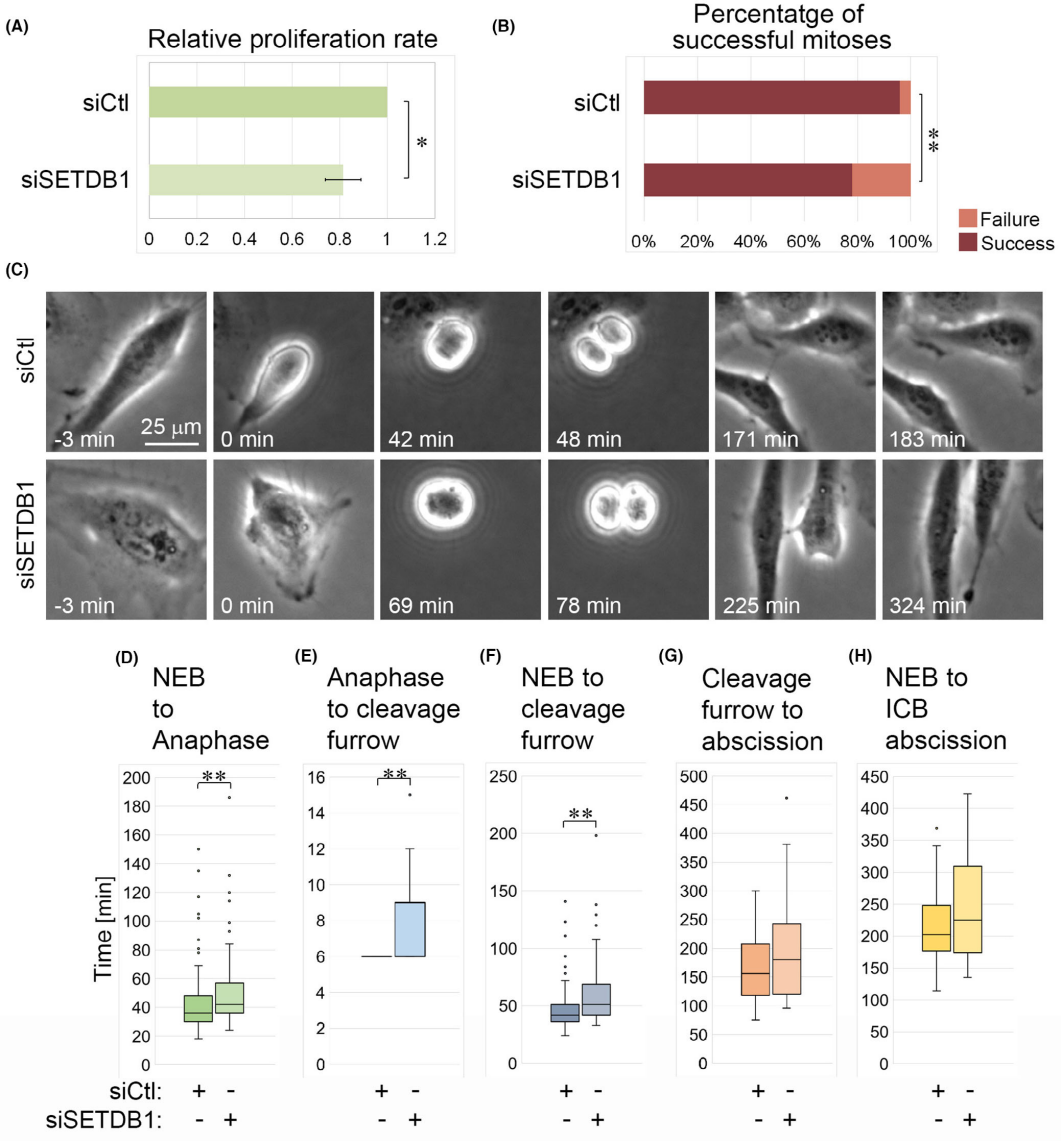
Reduced SETDB1 levels alter the duration of mitotic phases. (A) Relative proliferation rate of HeLa cells transfected with either Ctl siRNA or SETDB1 siRNA as measured by the XTT assay. The average of three repetitions ±*SE* is shown. Ctl siRNA was set as 1. Statistical significance was calculated with Student's *t*‐test, *p ˂ 0.05. (B) The percentage of successful and failed mitotic events in HeLa cells transfected with either Ctl siRNA or SETDB1 siRNA. Statistical significance was calculated with Student's *t*‐test, **p ˂ 0.01. (C) Micrographs showing mitotic progression of HeLa cells transfected with either Ctl siRNA or SETDB1 siRNA. Scale bar: 25 μm. (D–H) Time periods of the indicated phases during cell division as calculated from time‐lapse images of HeLa cells transfected with either Ctl siRNA or SETDB1 siRNA. Frames were taken every 3 min. In each experiment a minimum of 40 cells were tracked for each condition. The average values of a representative experiment out of three experiments are presented. Statistical significance was calculated with Wilcoxon–Mann–Whitney test, **p ˂ 0.01

The finding that SETDB1 regulates MT dynamics led us to test if SETDB1 is important for cell division, a process that is heavily dependent on MT organization. Indeed, reduced SETDB1 levels attenuated the proliferation of HeLa cells by 20% (Figure 6A), while increasing unsuccessful mitotic events from 4.1% to 22.1% (Figure 6B). Measuring the lengths of the different cell division stages in cells that were able to finish mitosis successfully revealed a significant increase in the duration of both early and late mitosis in SETDB1 siRNA transfected cells in comparison to Ctl siRNA‐transfected cells. In SETDB1 siRNA‐transfected cells, the durations from nuclear envelope breakdown (NEB) to anaphase and from anaphase to the appearance of a cleavage furrow were increased by 17% and 40%, respectively, in comparison to control cells (Figure 6C–H).

### 3.4 Catalytic‐dead SETDB1 can affect MT growth rate

**FIGURE 7:**
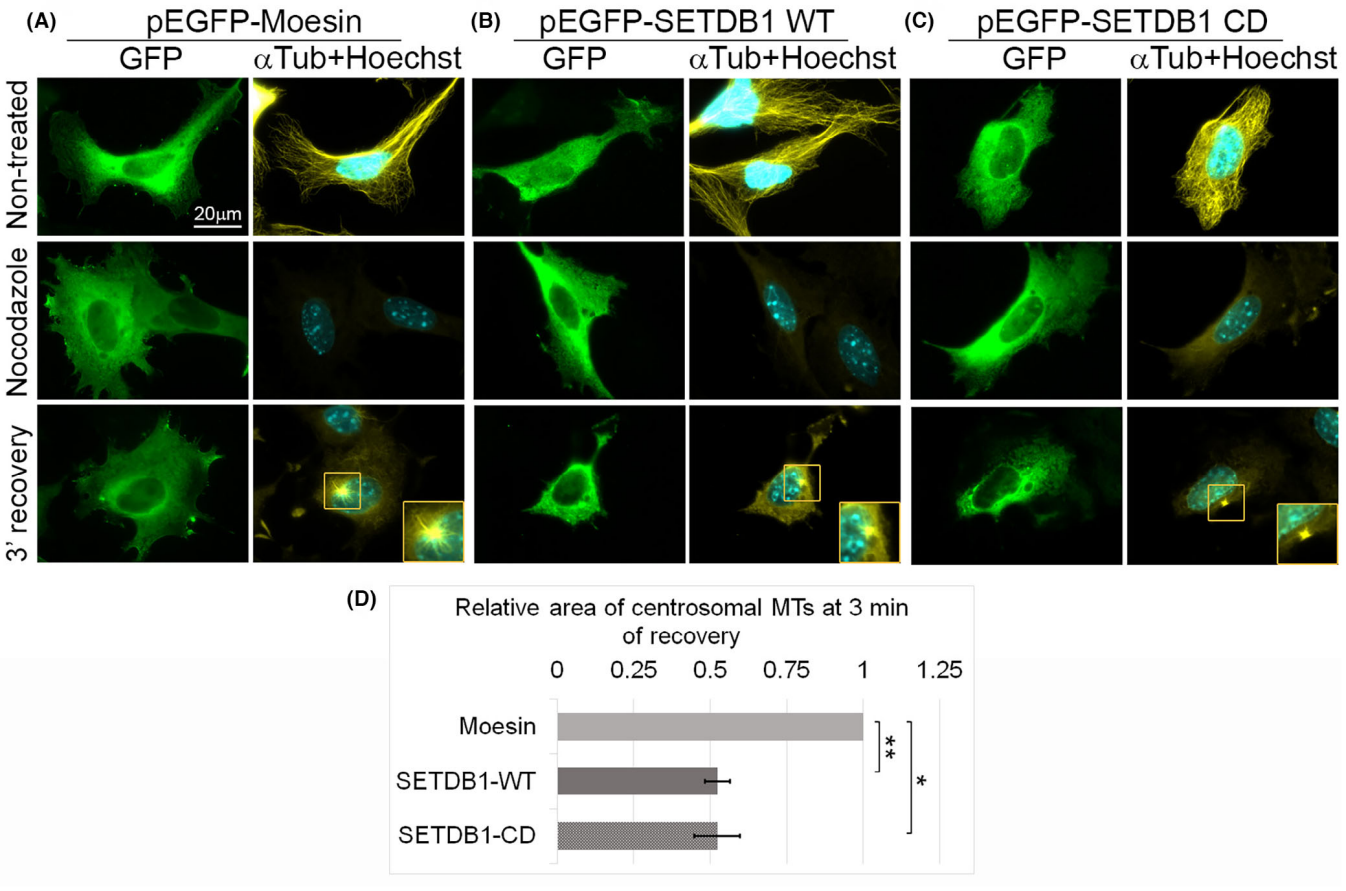
SETDB1 over‐expression enhances MT polymerization rate in KMT‐independent manner. (A–C) MTs were disrupted in B16‐F1 cells transfected with either pEGFP‐Moesin (A) or pEGFP‐SETDB1 WT (B) or pEGFP‐SETDB1 CD (C) by nocodazole treatment for 3 h. Following nocodazole removal, cells were further incubated for 3 min to allow MT polymerization. After fixation, cells were immunostained with antibodies against GFP and α‐tubulin and DNA was stained with Hoechst 33342. The MTOCs in the orange rectangles are magnified at the right bottom corner of the micrographs of recovery from nocodazole treatment. Scale bar: 20 μm. (D) Quantification of the recovery rate of MTs after nocodazole washout. The area covered by MTs around the MTOC was quantified by ImageJ software and normalized to the area in cells transfected with pEGFP‐Moesin. The bar graph shows the average relative area covered by MTs of three repetitions ±*SE*. At least 30 cells were measured for each condition in each repetition. Statistical significance was calculated with Student's *t‐*test, *p ˂ 0.05; **p ˂ 0.01

SETDB1 is a well‐established methyltransferase that was found to methylate both histones^4^ and non‐histone proteins.^19^, ^24^, ^40^ Moreover, recently α‐tubulin was found to be methylated at lysine 40 by SET domain containing 2 (SETD2).^41^ To evaluate if the effect of SETDB1 on MT growth rate is dependent on its methyltransferase activity, we repeated the MT recovery assay while over‐expressing either WT SETDB1 or H1224K point mutated SETDB1 (SETDB1‐CD) (Figure 6). The H1224K mutation was shown before to completely impair the methyltransferase activity of SETDB1.^4^ As shown in Figure 7, over‐expression of the CD SETDB1 attenuated MT recovery from nocodazole treatment to the same extent as over‐expression of WT SETDB1. Thus, SETDB1 could affect MT dynamics by a mechanism that is independent of its enzymatic activity.

### 3.5 SETDB1 affects tubulin acetylation

**FIGURE 8:**
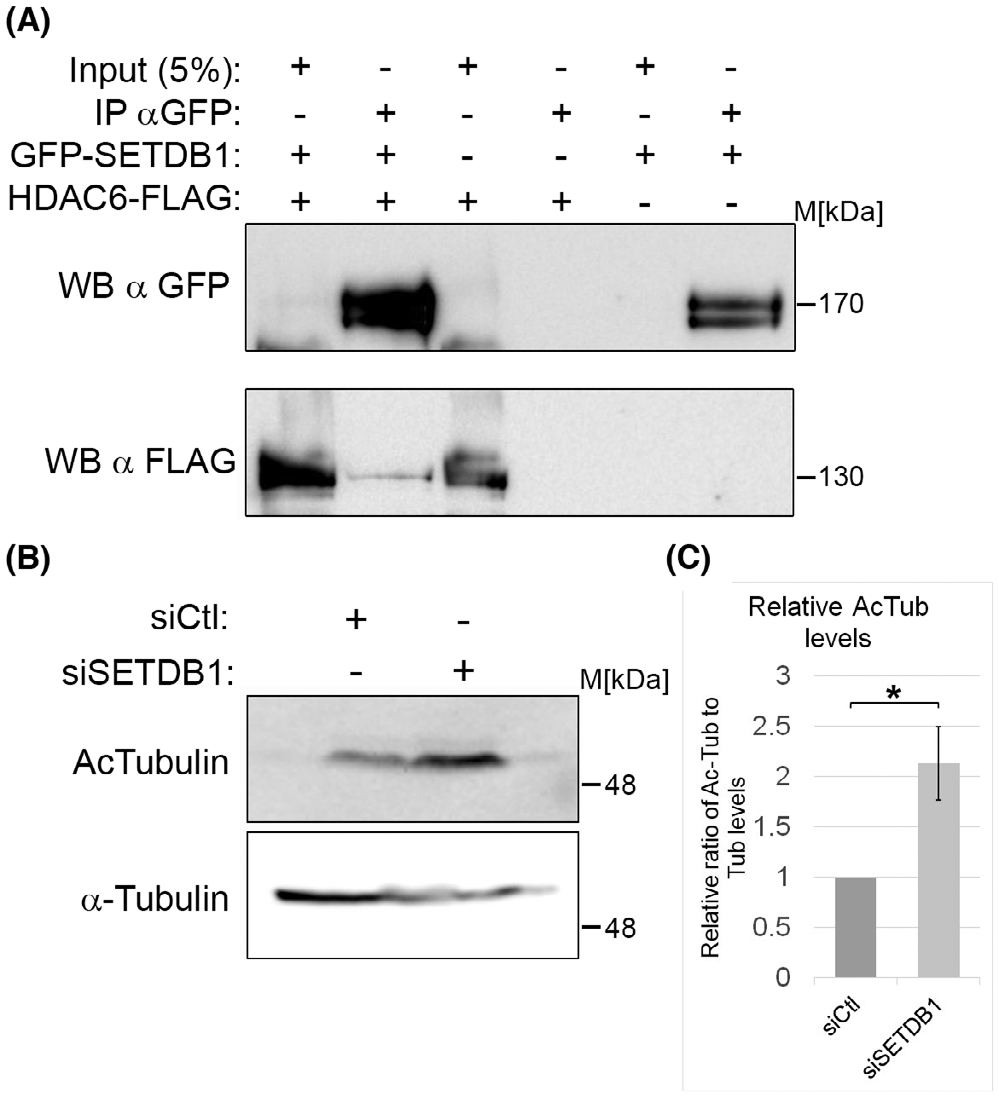
SETDB1‐HDAC6 interaction. (A) Co‐IP of over‐expressed SETDB1‐GFP and HDAC6‐FLAG in HEK293 cells. (B) Western blot analysis of the Ac‐tubulin levels in B16‐F1 cells transfected with either Ctl siRNA or siRNA against SETDB1. (C) The ratio of Ac‐tubulin to Tubulin levels in three repetitions normalized to the same ratio in Ctl siRNA‐transfected cells ±*SE*. Statistical significance was calculated with Student's *t‐*test, *p ˂ 0.05

The molecular mechanism by which SETDB1 affects MT dynamics seemed to be at least partially independent of its methyltransferase activity. To identify a molecular mechanism by which SETDB1 could affect MT dynamics, we took into account two features: the first is that nuclear SETDB1 is known to participate in a complex with HDAC1 and HDAC2 to repress transcription.^42^ The second is that acetylation of Lys40 in α‐tubulin (acetylated tubulin) is a key tubulin post‐translational modification, which is associated with stable MTs in various cellular contexts.^43^, ^44^ We hypothesized that SETDB1 may interact with a tubulin HDAC and affect its activity. Since the major tubulin deacetylase is HDAC6, we tested if SETDB1 can interact with it and affect tubulin acetylation levels. As shown in Figure 8A, HDAC6 co‐immunoprecipitated with SETDB1, suggesting an in vivo interaction between these two factors. In addition, reduction in SETDB1 levels led to an increase of 110% in the levels of tubulin acetylation (Figure 8B,C), thus supporting the hypothesis that SETDB1 may serve as a co‐factor of HDAC6 in the cytoplasm to support tubulin deacetylation to regulate MT dynamics.

## 4 DISCUSSION

SETDB1 is a well‐established H3K9 methyltransferase involved in embryogenesis and development^8^, ^14^, ^15^, ^16^, ^17^ as well as in the aetiology of cancer.^45^, ^46^, ^47^ Current perception is that SETDB1 affects these processes due to its nuclear localization, while any cytoplasmic localization of SETDB1 serves to either sequester it or methylate newly generated histone H3.^21^, ^22^, ^26^ Here we found that cytoplasm localized SETDB1 partially co‐localized with MTs and that SETDB1 co‐sedimented with MTs. Moreover, reduced SETDB1 levels altered MT dynamics: it sped up MT recovery from nocodazole treatment, and in steady‐state conditions it led to enhanced MT growth duration, length and rate in parallel to reduced frequency of catastrophe events. Notably, OE SETDB1 affected MT growth at the opposite direction: it led to slower MT recovery from nocodazole treatment. OE SETDB1 is mainly localized to the cytoplasm and hardly enters the nucleus as reported by others^22^, ^23^, ^39^ and also in our hands. Thus, the effect of OE SETDB1 on MT dynamics suggests that the impact of SETDB1 on MT growth rate is due to the activity of its cytoplasmic pool rather than the nuclear SETDB1.

Recently, the methyltransferases SETD2 and KMT5A were shown to methylate α‐tubulin on K40 and K311, respectively, while SETD2 activity was limited to the M phase.^41^, ^48^ To assess if SETDB1 may affect MT dynamics by its methyltransferase activity, we evaluated the effects of CD SETDB1 on MT dynamics and found that both WT and CD SETDB1 reduced MT recovery rate from nocodazole treatment. These results suggest that the effects of SETDB1 on MTs did not require its methyltransferase activity. This observation is in agreement with studies on SETDB1 oncogenic activity in the zebrafish model: over‐expression of SETDB1 was found to accelerate melanoma onset in a zebrafish model for melanoma formation and progression. Notably, over‐expression of the enzymatically inactive H1224K SETDB1 (CD SETDB1) still significantly accelerated melanoma onset, although to a lower extent than WT SETDB1.^27^ More recently, the *Caenorhabditis elegans* homologue of SETDB1, MET‐2, was shown to be able to affect transcription even when its methyltransferase activity was ablated.^49^ This suggests that the oncogenic function of SETDB1 is partially contributed by methyltransferase independent activities of the protein. One of these activities may be the modulation of MT dynamics.

Based on the findings that SETDB1 interacted with HDAC6 and that reduced levels of SETDB1 led to increased tubulin acetylation, we hypothesize that SETDB1 can modulate MT dynamics by affecting the activity of the tubulin deacetylase HDAC6. Tubulin acetylation is associated with more stable MTs (^43^, ^44^), increased mechanical stabilization of MTs and reduced MT mechanical breakage.^50^ Thus, SETDB1 may support HDAC6 activity to reduce MT stability, leading to increased MT catastrophe rate and slower recovery from nocodazole treatment. Notably, altered levels of both tubulin acetylation and HDAC6 were detected in several types of cancer. In some cases, such as in breast cancer and glioblastoma, HDAC6 was shown to support tumour progression.^51^

Our results, taken together with previous reports, suggest that SETDB1 may affect various aspects of tumour progression such as cell division and cell migration, at multiple levels by different mechanisms. At the chromatin level, it may affect transcriptional control of key factors to support cell migration and proliferation^28^, ^30^, ^52^, ^53^ and it may promote global chromatin condensation to support cell migration^52^, ^54^; while at the MT level, it may modulate MT dynamics possibly by affecting the activity and/or the binding of microtubule‐associated proteins (MAPs) to MTs such as HDAC6.

Cross‐talk between interphase chromatin and MTs was found before: MT‐driven mechanical forces were shown to alter chromatin organization,^55^, ^56^ the motor protein kinesin KIF4 was found to be involved in heterochromatin formation, transcription and DNA repair.^57^ Dynein light chain 1 (DLC1) was found to be involved in transcriptional control.^56^ Tau nuclear subcellular localization is thought to associate with DNA damage protection.^58^ LIS1, a regulator of cytoplasmic dynein, was shown to interact with histone H1 and MeCP1 and to affect the chromatin binding of the latter.^59^ Thus, SETDB1 seems to join this growing group of factors with activities in both the chromatin and the MT worlds.

## AUTHOR CONTRIBUTIONS

Conceptualization, G.G., R.H.V. and T.L.; methodology, R.H.V., J.S., T.L. and G.G.; investigation, R.H.V., J.S. and N.P.; formal analysis, R.H.V.; writing—original draft, G.G.; writing—review and editing, R.H.V., J.S., N.P., T.L. and G.G.; supervision; T.L. and G.G.; funding acquisition, G.G.

## FUNDING INFORMATION

The research was supported by the Israel Cancer Association (grant no. 202220036), Ariel University and Ariel Scientific Innovations.

## CONFLICT OF INTEREST

The authors declare that they have no conflict of interest.

## Supporting information


**Figure S1.** SETDB1 siRNA.

**Movie S1.** MT plus end tracking in Ctl siRNA‐transfected cells.

**Movie S2.** MT plus end tracking in SETDB1 siRNA‐transfected cells.

## Data Availability

No codes or sequencing datasets were generated in this study. All materials and protocols will be available upon request.

## References

[1] Mozzetta C , Boyarchuk E , Pontis J , Ait‐Si‐Ali S . Sound of silence: the properties and functions of repressive Lys methyltransferases. Nat Rev Mol Cell Biol. 2015;16:499‐513. doi:10.1038/nrm4029 26204160

[2] Torrano J , Al Emran A , Hammerlindl H , Schaider H . Emerging roles of H3K9me3, SETDB1 and SETDB2 in therapy‐induced cellular reprogramming. Clin Epigenetics. 2019;11:43. doi:10.1186/s13148-019-0644-y 30850015 PMC6408861

[3] Jurkowska RZ , Qin S , Kungulovski G , et al. H3K14ac is linked to methylation of H3K9 by the triple Tudor domain of SETDB1. Nat Commun. 2017;8:2057. doi:10.1038/s41467-017-02259-9 29234025 PMC5727127

[4] Schultz DC . SETDB1: a novel KAP‐1‐associated histone H3, lysine 9‐specific methyltransferase that contributes to HP1‐mediated silencing of euchromatic genes by KRAB zinc‐finger proteins. Genes Dev. 2002;16:919‐932. doi:10.1101/gad.973302 11959841 PMC152359

[5] Keniry A , Gearing LJ , Jansz N , et al. Setdb1‐mediated H3K9 methylation is enriched on the inactive X and plays a role in its epigenetic silencing. Epigenetics Chromatin. 2016;9:16. doi:10.1186/s13072-016-0064-6 27195021 PMC4870784

[6] Minkovsky A , Sahakyan A , Rankin‐Gee E , Bonora G , Patel S , Plath K . The Mbd1‐Atf7ip‐Setdb1 pathway contributes to the maintenance of X chromosome inactivation. Epigenetics Chromatin. 2014;7:12. doi:10.1186/1756-8935-7-12 25028596 PMC4099106

[7] Cuellar TL , Herzner A‐M , Zhang X , et al. Silencing of retrotransposons by SETDB1 inhibits the interferon response in acute myeloid leukemia. J Cell Biol. 2017;216:3535‐3549. doi:10.1083/jcb.201612160 28887438 PMC5674883

[8] Liu S , Brind'Amour J , Karimi MM , et al. Setdb1 is required for germline development and silencing of H3K9me3‐marked endogenous retroviruses in primordial germ cells. Genes Dev. 2014;28:2041‐2055. doi:10.1101/gad.244848.114 25228647 PMC4173156

[9] Matsui T , Leung D , Miyashita H , et al. Proviral silencing in embryonic stem cells requires the histone methyltransferase ESET. Nature. 2010;464:927‐931. doi:10.1038/nature08858 20164836

[10] Sharif J , Endo TA , Nakayama M , et al. Activation of endogenous retroviruses in Dnmt1 −/− ESCs involves disruption of SETDB1‐mediated repression by NP95 binding to hemimethylated DNA. Cell Stem Cell. 2016;19:81‐94. doi:10.1016/j.stem.2016.03.013 27151458

[11] Du D , Katsuno Y , Meyer D , et al. Smad3‐mediated recruitment of the methyltransferase SETDB1/ESET controls *Snail1* expression and epithelial–mesenchymal transition. EMBO Rep. 2018;19:135‐155. doi:10.15252/embr.201744250 29233829 PMC5757214

[12] Jiang Y , Jakovcevski M , Bharadwaj R , et al. Setdb1 histone methyltransferase regulates mood‐related behaviors and expression of the NMDA receptor subunit NR2B. J Neurosci. 2010;30:7152‐7167. doi:10.1523/JNEUROSCI.1314-10.2010 20505083 PMC2893142

[13] Karimi MM , Goyal P , Maksakova IA , et al. DNA methylation and SETDB1/H3K9me3 regulate predominantly distinct sets of genes, retroelements, and chimeric transcripts in mESCs. Cell Stem Cell. 2011;8:676‐687. doi:10.1016/j.stem.2011.04.004 21624812 PMC3857791

[14] Bilodeau S , Kagey MH , Frampton GM , Rahl PB , Young RA . SetDB1 contributes to repression of genes encoding developmental regulators and maintenance of ES cell state. Genes Dev. 2009;23:2484‐2489. doi:10.1101/gad.1837309 19884255 PMC2779743

[15] Dodge JE , Kang Y‐K , Beppu H , Lei H , Li E . Histone H3‐K9 methyltransferase ESET is essential for early development. Mol Cell Biol. 2004;24:2478‐2486. doi:10.1128/MCB.24.6.2478-2486.2004 14993285 PMC355869

[16] Jiang Y , Loh Y‐HE , Rajarajan P , et al. The methyltransferase SETDB1 regulates a large neuron‐specific topological chromatin domain. Nat Genet. 2017;49:1239‐1250. doi:10.1038/ng.3906 28671686 PMC5560095

[17] Takikita S , Muro R , Takai T , et al. A histone methyltransferase ESET is critical for T cell development. J Immunol. 2016;197:2269‐2279. doi:10.4049/jimmunol.1502486 27511731

[18] Binda O , LeRoy G , Bua DJ , Garcia BA , Gozani O , Richard S . Trimethylation of histone H3 lysine 4 impairs methylation of histone H3 lysine 9: regulation of lysine methyltransferases by physical interaction with their substrates. Epigenetics. 2010;5:767‐775. doi:10.4161/epi.5.8.13278 21124070 PMC3052887

[19] Fei Q , Shang K , Zhang J , et al. Histone methyltransferase SETDB1 regulates liver cancer cell growth through methylation of p53. Nat Commun. 2015;6:8651. doi:10.1038/ncomms9651 26471002 PMC5426523

[20] Hwang YJ , Han D , Kim KY , et al. ESET methylates UBF at K232/254 and regulates nucleolar heterochromatin plasticity and rDNA transcription. Nucleic Acids Res. 2014;42:1628‐1643. doi:10.1093/nar/gkt1041 24234436 PMC3919562

[21] Beyer S , Pontis J , Schirwis E , et al. Canonical Wnt signalling regulates nuclear export of Setdb1 during skeletal muscle terminal differentiation. Cell Discovery. 2016;2:16037. doi:10.1038/celldisc.2016.37 27790377 PMC5067623

[22] Cho S , Park JS , Kang Y‐K . Regulated nuclear entry of over‐expressed Setdb1. Genes Cells. 2013;18:694‐703. doi:10.1111/gtc.12068 23782009

[23] Tachibana K , Gotoh E , Kawamata N , et al. Analysis of the subcellular localization of the human histone methyltransferase SETDB1. Biochem Biophys Res Commun. 2015;465:725‐731. doi:10.1016/j.bbrc.2015.08.065 26296461

[24] Wang G , Long J , Gao Y , et al. SETDB1‐mediated methylation of Akt promotes its K63‐linked ubiquitination and activation leading to tumorigenesis. Nat Cell Biol. 2019;21:214‐225. doi:10.1038/s41556-018-0266-1 30692626 PMC6414065

[25] Kostaki M , Manona AD , Stavraka I , et al. High‐frequency *p16* ^ *INK 4A* ^ promoter methylation is associated with histone methyltransferase SETDB1 expression in sporadic cutaneous melanoma. Exp Dermatol. 2014;23:332‐338. doi:10.1111/exd.12398 24673285

[26] Loyola A , Bonaldi T , Roche D , Imhof A , Almouzni G . PTMs on H3 variants before chromatin assembly potentiate their final epigenetic state. Mol Cell. 2006;24:309‐316. doi:10.1016/j.molcel.2006.08.019 17052464

[27] Ceol CJ , Houvras Y , Jane‐Valbuena J , et al. The histone methyltransferase SETDB1 is recurrently amplified in melanoma and accelerates its onset. Nature. 2011;471:513‐517. doi:10.1038/nature09806 21430779 PMC3348545

[28] Orouji E , Federico A , Larribère L , et al. Histone methyltransferase SETDB1 contributes to melanoma tumorigenesis and serves as a new potential therapeutic target. Int J Cancer. 2019;145:3462‐3477. doi:10.1002/ijc.32432 31131878

[29] Chen K , Zhang F , Ding J , et al. Histone methyltransferase SETDB1 promotes the progression of colorectal cancer by inhibiting the expression of TP53. J Cancer. 2017;8:3318‐3330. doi:10.7150/jca.20482 29158805 PMC5665049

[30] Yu L , Ye F , Li Y‐Y , et al. Histone methyltransferase SETDB1 promotes colorectal cancer proliferation through the STAT1‐CCND1/CDK6 axis. Carcinogenesis. 2019;41:678‐688. doi:10.1093/carcin/bgz131 31306481

[31] Zhang Y , Huang J , Li Q , et al. Histone methyltransferase SETDB1 promotes cells proliferation and migration by interacting withTiam1 in hepatocellular carcinoma. BMC Cancer. 2018;18:539. doi:10.1186/s12885-018-4464-9 29739365 PMC5941371

[32] Rodriguez‐Paredes M , Martinez de Paz A , Simó‐Riudalbas L , et al. Gene amplification of the histone methyltransferase SETDB1 contributes to human lung tumorigenesis. Oncogene. 2014;33:2807‐2813. doi:10.1038/onc.2013.239 23770855 PMC4031636

[33] Sun Q‐Y , Ding L‐W , Xiao J‐F , et al. SETDB1 accelerates tumourigenesis by regulating the WNT signalling pathway. J Pathol. 2015;235:559‐570. doi:10.1002/path.4482 25404354 PMC4333197

[34] Kang Y‐K . Surveillance of retroelement expression and nucleic‐acid immunity by histone Methyltransferase SETDB1. Bioessays. 2018;40:1800058. doi:10.1002/bies.201800058 29897144

[35] Fritsch L , Robin P , Mathieu JRR , et al. A subset of the histone H3 lysine 9 methyltransferases Suv39h1, G9a, GLP, and SETDB1 participate in a multimeric complex. Mol Cell. 2010;37:46‐56. doi:10.1016/j.molcel.2009.12.017 20129054

[36] Kawaguchi Y , Kovacs JJ , McLaurin A , Vance JM , Ito A , Yao TP . The deacetylase HDAC6 regulates aggresome formation and cell viability in response to misfolded protein stress. Cell. 2003;115:727‐738. doi:10.1016/s0092-8674(03)00939-5 14675537

[37] Zheng P , Obara CJ , Szczesna E , et al. ER proteins decipher the tubulin code to regulate organelle distribution. Nature. 2022;601:132‐138. doi:10.1038/s41586-021-04204-9 34912111 PMC8732269

[38] Meijering E , Dzyubachyk O , Smal I . Methods for cell and particle tracking. Methods in Enzymology. Elsevier; 2012:183‐200. 10.1016/B978-0-12-391857-4.00009-4 22264535

[39] Tsusaka T , Shimura C , Shinkai Y . ATF7IP regulates SETDB1 nuclear localization and increases its ubiquitination. EMBO Rep. 2019;20:e48297. doi:10.15252/embr.201948297 31576654 PMC6893292

[40] Guo J , Dai X , Laurent B , et al. AKT methylation by SETDB1 promotes AKT kinase activity and oncogenic functions. Nat Cell Biol. 2019;21:226‐237. doi:10.1038/s41556-018-0261-6 30692625 PMC6377565

[41] Park IY , Powell RT , Tripathi DN , et al. Dual chromatin and cytoskeletal remodeling by SETD2. Cell. 2016;166:950‐962. doi:10.1016/j.cell.2016.07.005 27518565 PMC5101839

[42] Yang L , Mei Q , Zielinska‐Kwiatkowska A , et al. An ERG (ets‐related gene)‐associated histone methyltransferase interacts with histone deacetylases 1/2 and transcription co‐repressors mSin3A/B. Biochem J. 2003;369:651‐657. doi:10.1042/bj20020854 12398767 PMC1223118

[43] Mu A , Latario CJ , Pickrell LE , Higgs HN . Lysine acetylation of cytoskeletal proteins: emergence of an actin code. J Cell Biol. 2020;219:e202006151. doi:10.1083/jcb.202006151 33044556 PMC7555357

[44] Li L , Yang X‐J . Tubulin acetylation: responsible enzymes, biological functions and human diseases. Cell Mol Life Sci. 2015;72:4237‐4255. doi:10.1007/s00018-015-2000-5 26227334 PMC11113413

[45] Batham J , Lim PS , Rao, S . SETDB‐1: a potential epigenetic regulator in breast cancer metastasis. Cancer. 2019;11:1143. doi:10.3390/cancers11081143 PMC6721492 31405032

[46] Karanth AV , Maniswami RR , Prashanth S , et al. Emerging role of SETDB1 as a therapeutic target. Expert Opin Ther Targets. 2017;21:319‐331. doi:10.1080/14728222.2017.1279604 28076698

[47] Strepkos D , Markouli M , Klonou A , Papavassiliou AG , Piperi C . Histone methyltransferase SETDB1: a common denominator of tumorigenesis with therapeutic potential. Cancer Res. 2021;81:525‐534. doi:10.1158/0008-5472.CAN-20-2906 33115801

[48] Chin HG , Esteve P‐O , Ruse C , et al. The microtubule‐associated histone methyltransferase SET8, facilitated by transcription factor LSF, methylates α‐tubulin. J Biol Chem. 2020;295:4748‐4759. doi:10.1074/jbc.RA119.010951 32111740 PMC7135998

[49] Delaney CE , Methot SP , Kalck V , et al. SETDB1‐like MET‐2 promotes transcriptional silencing and development independently of its H3K9me‐associated catalytic activity. Nat Struct Mol Biol. 2022;29:85‐96. doi:10.1038/s41594-021-00712-4 35102319 PMC8850192

[50] Xu Z , Schaedel L , Portran D , et al. Microtubules acquire resistance from mechanical breakage through intralumenal acetylation. Science. 2017;356:328‐332. doi:10.1126/science.aai8764 28428427 PMC5457157

[51] Trisciuoglio D , Degrassi F . The tubulin code and tubulin‐modifying enzymes in autophagy and cancer. Cancers (Basel). 2021;14:6. doi:10.3390/cancers14010006 35008169 PMC8750717

[52] Gerlitz G . The emerging roles of heterochromatin in cell migration. Front Cell Dev Biol. 2020;8:394. doi:10.3389/fcell.2020.00394 32528959 PMC7266953

[53] Zakharova VV , Magnitov MD , Del Maestro L , et al. SETDB1 fuels the lung cancer phenotype by modulating epigenome, 3D genome organization and chromatin mechanical properties. Nucleic Acids Res. 2022;50:4389‐4413. doi:10.1093/nar/gkac234 35474385 PMC9071401

[54] Maizels Y , Elbaz A , Hernandez‐Vicens R , Sandrusy O , Rosenberg A , Gerlitz G . Increased chromatin plasticity supports enhanced metastatic potential of mouse melanoma cells. Exp Cell Res. 2017;357:282‐290. doi:10.1016/j.yexcr.2017.05.025 28551377

[55] Gerlitz G , Reiner O , Bustin M . Microtubule dynamics alter the interphase nucleus. Cell Mol Life Sci. 2013;70:1255‐1268. doi:10.1007/s00018-012-1200-5 23117601 PMC11113956

[56] Maizels Y , Gerlitz G . Shaping of interphase chromosomes by the microtubule network. FEBS Journal. 2015;282:3500‐3524. doi:10.1111/febs.13334 26040675

[57] Mazumdar M , Sung M‐H , Misteli T . Chromatin maintenance by a molecular motor protein. Nucleus. 2011;2:591‐600. doi:10.4161/nucl.2.6.18044 22130187 PMC3324347

[58] Diez L , Wegmann S . Nuclear transport deficits in tau‐related neurodegenerative diseases. Front Neurol. 2020;11:105. doi:10.3389/fneur.2020.01056 33101165 PMC7546323

[59] Keidar L , Gerlitz G , Kshirsagar A , et al. Interplay of LIS1 and MeCP2: interactions and implications with the neurodevelopmental disorders Lissencephaly and Rett syndrome. Front Cell Neurosci. 2019;13:370. doi:10.3389/fncel.2019.00370 31474834 PMC6703185

