# Supplementary material for: SETDB1 regulates microtubule dynamics
Source: Cell Prolif. 2022 Nov 3;55(12):e13348. doi: 10.1111/cpr.13348 (PMC9715361; doi:10.1111/cpr.13348)
Supplement: Supplementary file 1 — Figure S1. SETDB1 siRNA. [file CPR-55-e13348-s001.docx]

**SETDB1 regulates microtubule dynamics**

Rosari Hernandez-Vicens^1^, Jagreeti Singh^1^, Nomi Pernicone^1^, Tamar Listovsky^1,2,3^ and Gabi Gerlitz^1,2,^*

**Supplemental materials**

**
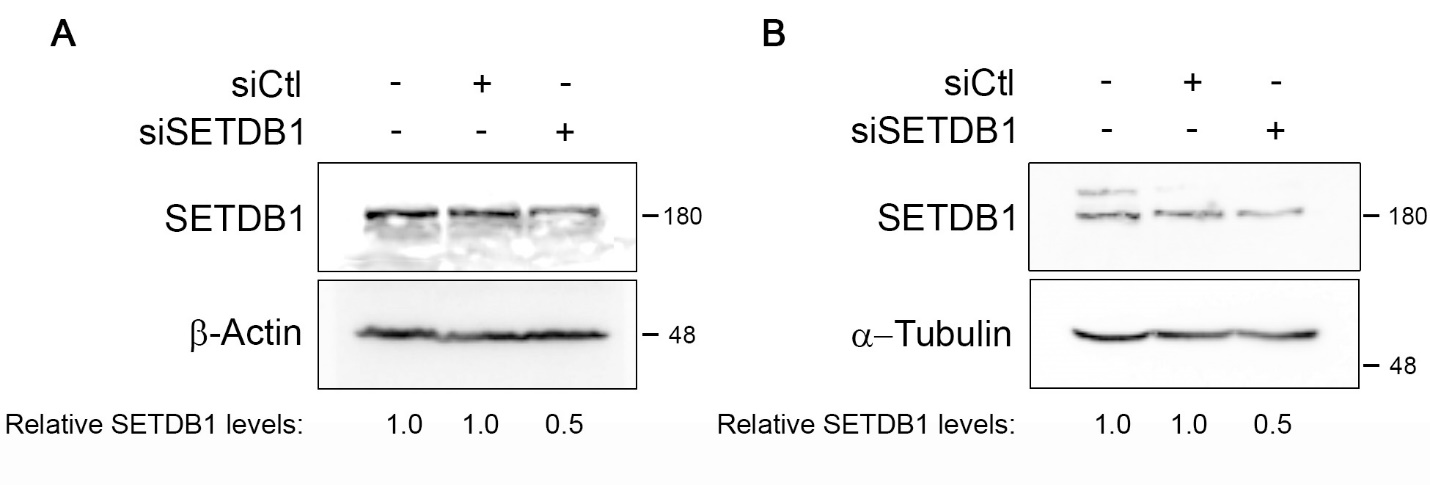
Sup Figure 1. SETDB1 siRNA.**

SETDB1 protein levels in mouse B16-F1 cells (A) and in human WM266.4 (B) after treatment with Ctl siRNA or SETDB1 siRNA. SETDB1 levels were normalized to β-Actin or α-Tubulin and the ratio for non-transfected cells was set as one.

**Sup Movie 1. MT plus end tracking in Ctl siRNA-transfected cells**

HeLa cells co-transfected with Ctl siRNA and EB1-GFP vector were placed in an environmental chamber and observed with an Olympus 1X81 fluorescent microscope. The cells were imaged every 3 seconds for 1.5 minutes. The movie that was generated by ImageJ presents 4 frames per second. Scale bar: 10 μm.

**Sup Movie 2. MT plus end tracking in SETDB1 siRNA-transfected cells**

HeLa cells co-transfected with SETDB1 siRNA and EB1-GFP vector were placed in an environmental chamber and observed with an Olympus 1X81 fluorescent microscope. The cells were imaged every 3 seconds for 1.5 minutes. The movie that was generated by ImageJ presents 4 frames per second. Scale bar: 10 μm.
